# Role of Acute Normovolemic Hemodilution in Pregnancy for Emergency Cesarean Section in a Patient With Bombay Blood Group

**DOI:** 10.7759/cureus.37995

**Published:** 2023-04-22

**Authors:** Protiti Chatterjee, Karthikeyan Alagumalai, Sakthirajan Panneerselvam

**Affiliations:** 1 Anaesthesiology and Critical Care, Jawaharlal Institute of Postgraduate Medical Education and Research, Puducherry, IND

**Keywords:** perioperative blood management, postpartum hemorrhage, bombay blood group, emergency cesarean section, acute normovolemic hemodilution

## Abstract

Postpartum hemorrhage (PPH) is among the leading causes of maternal morbidity and mortality, and various blood conservation strategies can be implemented to minimize blood loss. Acute normovolemic hemodilution (ANH) is a simple yet effective blood management tool in the armamentarium of an anesthesiologist, the use of which can be considered for patients undergoing surgical procedures with inherent bleeding risks, procedures where more than 50% of the patient’s circulating blood volume is lost, patients with multiple antibodies and rare blood groups, and those unwilling to undergo an allogenic blood transfusion. We hereby describe the performance of ANH in a pregnant woman with a Bombay blood group during an emergency cesarean section. Existing literature on ANH in obstetric patients does not report adverse fetal or maternal outcomes due to preoperative blood donation and advocates its selected use in situations where the benefits outweigh the risks.

## Introduction

Postpartum hemorrhage (PPH) is one of the leading causes of maternal morbidity and mortality. It is estimated that maternal mortality due to postpartum hemorrhage ranges from 6% in developed countries to 34% in developing countries [1]. Various blood conservation strategies can be employed to minimize this blood loss and reduce the need for allogeneic blood transfusions, like preoperative autologous blood donation (PABD), acute normovolemic hemodilution (ANH), and intraoperative blood salvage (IBS). PABD, wherein blood collected from patients in the preoperative period is transfused during surgery, carries an attendant risk of anemia due to preoperative blood donation, does not eliminate the risks associated with transfusion, cannot be used during emergencies, and is not acceptable to Jehovah’s Witnesses [2]. ANH is the process of extracting a predetermined volume of blood before the surgical incision while maintaining euvolemia with colloids or crystalloid supplementation. During the process, 1-3 units of blood are removed from the patient, targeting a postprocedural hemoglobin of 8-9 g/dl. The actual volume of blood withdrawn varies for each patient and depends on factors such as estimated total blood volume, baseline hemoglobin, hemodynamic stability, and anticipated blood loss during the surgery. ANH can be considered for patients undergoing surgical procedures with inherent bleeding risks, procedures where more than 50% of the patient’s circulating blood volume is lost, patients with multiple antibodies, those with rare blood groups, and those not willing to undergo allogenic blood transfusion [3]. ANH provides fresh autologous blood and is the least expensive among the various autologous blood procurement methods [4].

## Case presentation

A 31-year-old primigravida at 38+1 weeks of gestation was posted for emergency lower segment cesarean section (LSCS) in view of non-progressive labor. Her co-morbidities included hypothyroidism, gestational diabetes mellitus, gestational hypertension, and bronchial asthma. Her preoperative investigations showed a hemoglobin of 10.8 g/dl, and she was found to belong to the Bombay blood group. Her airway was anticipated to be difficult in view of Modified Mallampati grade 3 and co-existing morbid obesity with a body mass index (BMI) of 39 kg/m2. Other investigations were within normal limits, and her hemodynamic parameters were stable. The patient was deemed to be at high risk for PPH given her 20-hour oxytocin induction, hypothyroidism, and morbid obesity. Intraoperative ANH was planned for her, given the increased risks of hemorrhage and the non-availability of compatible blood products at that point in time.

Under aseptic precautions, ANH was performed by extracting 350 ml of blood, which was simultaneously replaced with crystalloids to maintain normovolemia. The formula used for calculating the maximum allowable blood volume to be extracted was [5]:

V = EBV x ({Hgb-i - Hgb-f} ÷ Hgb-av)

where V is the volume of blood to be removed (liters), EBV is the baseline estimated blood volume (liters) of the patient, Hgb-i is the initial Hgb (g/dL), Hgb-f is the final desired Hgb (g/dL), and Hgb-av is the average Hgb (the average of Hgb-i and Hgb-f). Assuming a final hemoglobin value of 9 g/dl and an estimated blood volume of 60 ml/kg, the total blood volume that could be withdrawn totaled 750 ml. Accordingly, half of the allowable volume was removed, keeping a safety margin in the event of acute blood loss during LSCS.

A weighing scale was used to measure the weight of the blood-collecting bag containing citrate-phosphate-dextrose-adenine (CPDA) buffer, and blood was collected up to an additional 450 g. Gentle agitation of the bag was done frequently to ensure proper mixing of the blood and anticoagulant. After adequately preloading the patient with crystalloids, spinal anesthesia was administered with 2 ml of 0.5% bupivacaine, and LSCS was performed. Blood loss during the surgery totaled 800 ml. Using topical hemostatic agents, the surgeons achieved meticulous hemostasis. Postoperatively, the withdrawn blood was transfused back to the patient. The patient was hemodynamically stable during the perioperative period, and the rest of her stay in the hospital was uneventful.

## Discussion

The Bombay blood group, otherwise known as the h/h or Oh group, is seen in about one in 10,000 individuals in India and is one of the rarest blood groups found [6]. Patients with this blood group are devoid of A, B, or H antigens in their cells and are known to have high titers of anti-H antibodies, which can result in significant fetal and maternal hemolysis if blood other than the Bombay phenotype is transfused [7]. Literature on patients with the Bombay blood group is scarce, with a few case reports describing the management of such patients during pregnancy. The Bombay phenotype poses unique challenges during pregnancy, especially when the risk of peripartum hemorrhage is high, as was the case in our patient. At the outset, blood grouping on short notice for such patients may be difficult, and reference laboratories may be required for confirmation of the blood group. In such cases, blood grouping early in pregnancy may aid in pre-emptive planning for autologous donation [8].

**Figure 1:**
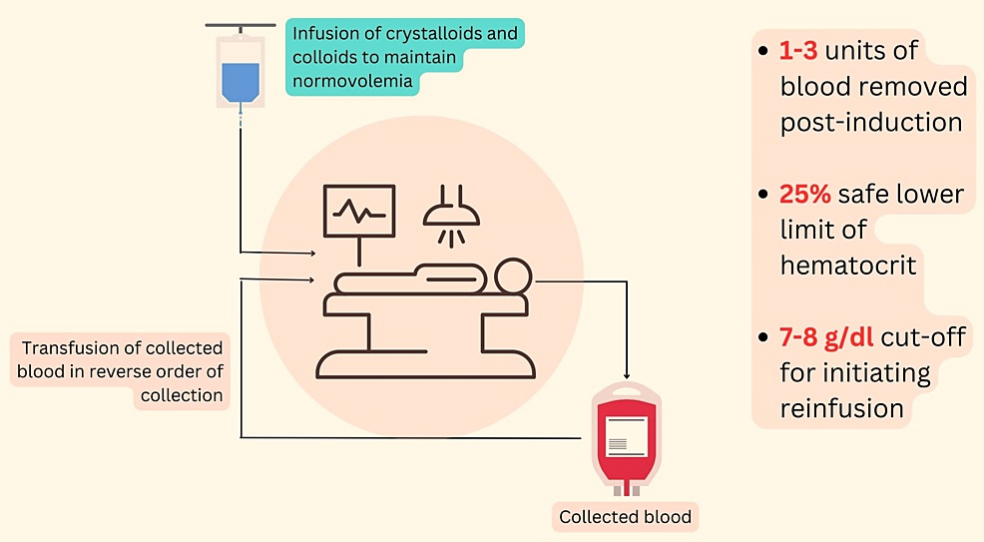
Acute normovolemic hemodilution: facts and figures

ANH is a safe and feasible blood management strategy for parturients undergoing cesarean sections who are at an elevated risk for bleeding. Studies have shown that the predonation of blood by pregnant women for autologous transfusion does not lead to adverse fetal outcomes [9]. An infographic on the salient facts pertaining to ANH has been provided below (Figure 1).

In this case, performing ANH was beneficial because of her high risk of PPH, the lack of availability of compatible blood in the blood bank, and the emergency nature of her surgery. Had ANH not been performed, PPH could have led to disastrous sequelae such as hypovolemic shock, severe anemia, hemodynamic instability requiring vasopressor support, coagulopathy, mechanical ventilation, cardiac compromise, and a prolonged ICU stay [10].

With the limited available evidence, widespread routine use of ANH for elective procedures is currently not recommended [11]. There is a need for large, controlled, prospective, randomized clinical trials to investigate both the risks and benefits of this technique. However, in select clinical situations where blood salvaging strategies are necessary and where the benefits outweigh the inherent risks, ANH can be performed to reduce maternal morbidity and mortality.

## Conclusions

Among the existing blood salvaging strategies, ANH offers myriad benefits. The procedure is easy to perform, incurs almost no added expense, carries no risks of transmitting blood-borne infections, minimizes the risks of allergy, and is admissible for Jehovah’s Witnesses.

Literature on the performance of ANH in pregnant women reports no increased fetal or maternal adverse outcomes, and hence, it is considered relatively safe. However, large-scale randomized clinical trials are warranted to investigate the attendant risks and benefits of the procedure. Moreover, data on parturients with the Bombay blood group is scarce, with few case reports describing their management during pregnancy. The rarity of our patient’s blood group, the high risk of PPH, and the emergency nature of surgery prompted us to implement ANH in order to avert the potentially disastrous aftermath of PPH.
